# Hospital Meetings, &c.

**Published:** 1900-08-18

**Authors:** 


### HOSPITAL MEETINGS, &C.

### NATIONAL HOSPITAL FOR THE PARALYSED AND EPILEPTIC.

A special general meeting of the National Hospital for the Paralysed and Epileptic was held at the hospital, Queen Square, on Saturday last, 11th inst., to consider whether the demand should be granted of the medical staff for direct representation on the board of management; and whether it was desirable to hold further inquiry into the condition of the hospital.

Mr. George W. E. Russell presided, and in opening the proceedings said a letter had been received from the Lord Chancellor regretting his inability to be present and take the chair that day, but expressing great goodwill towards the hospital, and his readiness to serve it in any way he could. The Chairman then called attention to the rules providing that only governors might vote at the meeting, but asking the indulgence of those present under existing circumstances should any member of the staff, not a governor or subscriber, wish to speak. He felt some apology was necessary with regard to the period of the year at which the meeting had been called, but claimed that the responsibility did not rest with the board, but with the staff. Their memorandum, though dated May, 1900, was not issued till June 16th. The board's reply was sent out on July 21st, the interval being occupied by the inquiry of the Special Committee appointed to look into the management. The letter of the staff in the *Times* of July 28th was the first statement that the differences between them were irreconcilable. After that the board would have felt that they were to blame had they allowed any time to be wasted before summoning a meeting of governors for the purpose of having the question argued out. It would be observed that there were two entirely distinct questions before them that day for decision. Question "A" was whether rule 5 of Section V. should be altered so as to allow members of the medical staff to sit on the board of management. Question "B" was whether it was desirable to hold a further inquiry into the condition of the hospital. He would deal with the latter first. He himself, though not connected with the institution so long as some members of the board, had had a seat on that board for about ten years. During the whole of that time he had made it a practice to visit the wards almost every week, and to cultivate and maintain friendly relations with the patients, both past and present. He had presided over the Special Committee, and their inquiry had been of a most thorough and exhaustive nature. The result of that examination into the working and arrangements of the hospital was that in the opinion of the board no further inquiry was necessary. (Hear, hear.) They could not say—and he thought it could not be said of any hospital — that no improvement was possible in any detail; but he could honestly say that such faults as they had discovered were infinitesimal.

But, while it must be pointed out that a further inquiry, if carried out by a legal body from outside, would be a costly affair, and must, to some extent, affect the working of the hospital, yet if the governors thought it necessary the board would offer no kind of opposition; but, on the contrary, would be eager to afford every possible facility. They had nothing to fear from the closest examination or from the widest publicity. But on the other matter—Question "A"—the attitude of the board was extremely different. They were deeply convinced that the demand of the medical staff for direct representation on the board of management must be resisted. The rule was one passed twenty years ago, and it was one which Mr. Chandler, to whom the hospital owed so much, was a party to. It was a rule in force at the London Hospital, at St. Bartholomew's, Guy's, the Middlesex, Brompton, London Fever, and the City of London Hospital for Diseases of the Chest; so that it could not be argued that their system was unique, or even exceptional. And it was based upon what they considered sound reasons. It was always possible that the exercise of some of the functions of the medical staff might call for discipline, and if so, it would be very inconvenient, to say the least, that they should be members of the tribunal which had to adjudicate upon them. As illustrations of the class of subject that might come before them he might mention the question of regularity of attendance, leave of absence and the provision of substitutes, and the admission of patients suitable for treatment. With regard to this last it might be urged that the medical man was the best judge, but he would remind them that under the Lunacy Laws it was exceedingly perilous to harbour persons of unsound mind; and by one of their rules it was expressly forbidden, even though the condition of a patient were such as to justify a medical man in saying he would be benefited by admission. Again, there were questions of complaint that might arise of neglect and so forth, in which a patient might appeal to the board. It seemed to him quite obvious that the medical men whose conduct might be called in question should not sit on the tribunal whose duty it was to try them. But although the board could not be parties to the admission of the staff to the governing body of the hospital, they felt most strongly that the greatest possible benefit would arise from a free interchange of opinion between them, and for that purpose the medical council existed. No medical matter was dealt with without reference to the Medical Committee, and all its representations were regarded with careful and courteous consideration. He knew of no instance in which any recommendation had been disregarded or treated lightly. In fact, the staff's own memorandum bore this out, for under the heading "Diet of Patients" it was expressly stated that their recommendations had been sanctioned and adopted by the board. So they had on one side and the other a recognised body; and between them, as a medium of communication, was the secretary-director. It was due to him to say that, despite the present trouble, there was no general feeling of hostility towards him; and though it could not be disputed that in some instances relations were strained, yet, on the whole, they were of a courteous and amicable character. The board considered that that office had been most ably and efficiently filled by Mr. Burford Rawlings. At one time they had had a system by which delegates were sent by the medical staff to the board when medical matters were under consideration, and the board regretted that that system had been discontinued, for he freely admitted that in some cases personal communication was better than that through an intermediary. The demand for direct representation on the board only became acute in the spring of last year—1899—and when that claim was first urged it was not on the ground that the patients were suffering neglect; the sole cause was that the board had recently introduced the system of payment by out-patients. In passing, he was bound to say a word of justification for that step. The income of the hospital did not increase by leaps and bounds, while the outgoings in some departments were perpetually increasing. This was mainly due to alterations in treatment and diet. As to that their staff were probably the best judges in Europe, and their decisions were entitled to absolute respect. But these alterations in diet greatly increased their housekeeping expenses, and these did advance by leaps and bounds. In considering this it occurred to the board that a very convenient and harmless way of adding to their income was, not to compel, but to invite, such of their out-patients as could afford it to contribute towards the cost of the medicines supplied to them—not for the medical attendance at all. They suggested that in the case of some patients, not rich but not abjectly poor, it might be a relief to their minds if they had an opportunity of doing so. In practice the system had been found to work well. It had yielded an increase of between £800 and £900 a year; it had proved no hardship; the class of patients had not altered at all; and they welcomed the opportunity of making some slight return for the benefits received. However, last year the medical staff thought it right to bring the matter before the Sunday Fund with a view to the suspension of the usual grant because of those payments. But when the staff sent out their statement, dated May last, they based their claim to admission on the board solely on the ground that the patients were suffering from certain abuses which the presence of medical men on the board would remedy. Considering this claim, he wished to draw particular attention to the following point: In the interests of the patients in the hospital, and in the interests of science as well, any shortcoming should have been reported directly to those in authority. But, in truth, the staff had never, down to June 16th last, suggested that the domestic condition of the hospital was other than satisfactory. These charges could not therefore possibly be well founded, for if they were they would throw a very invidious light on those who thus allowed abuses to exist without redress during a considerable period of time. He could not think they would have allowed them to exist without calling attention to them. But, admitting, for the sake of argument, what he did not admit in fact, was it to be thought that gentlemen who had so allowed mischief and evils to exist unremedied would be a valuable accession to the practical management of an institution which required promptness and business ability? The fact that they had never made any such representations was enough in itself to dispose of their charges. And if any further refutation were necessary, it was forthcoming in the evidence of Mr. Sydney Holland and Sir Henry Burdett, than whom none better knew what hospital requirements were, and who, after a careful examination, expressed themselves as perfectly satisfied in every detail. In addition to that again, they had the fact that the doctors had continued to send patients to the hospital, and also to communicate with the board on matters as they arose, although their letter to the *Times* of the 8th inst. would lead one to suppose that communications had quite ceased. He thought they had grave cause of complaint with regard to the question of the Hospital Sunday Fund. The medical staff had used their best endeavours to secure the suspension of the usual grant, and that he considered was not legitimate warfare; it seemed to him to be highly unfriendly and injurious to the hospital itself. He believed, however, that the grant was only suspended, and when the Distribution Committee of the Fund saw the case of the board as set forth in pages 8, 9, and 10 of their memorandum, he felt sure they would reconsider their decision. The speaker then referred to the prejudice which existed in some quarters against vivisection, and expressed the opinion that if gentlemen recognised as prominent vivisectors were known to be on the governing body of the hospital it might operate adversely with regard to their subscription list, despite the fact that the hospital possessed no medical school or physiological laboratory. In conclusion, after observing that, with the most trifling exceptions, the board had been during the many years the work had been carried on, on the most friendly terms with the medical staff, and were firmly convinced of their humanity, honour, and uprightness; the work they were mutually engaged in was one essentially of Christian philanthropy, and carried on in the sole interest of suffering humanity, he moved, "That it is inexpedient to grant the demand of the medical and surgical staff for direct representation upon the board."

The resolution was seconded by Mr. Hopwood, C.B., C.M.G., of the Railway Department of the Board of Trade, and supported by Mr. Bowyer and by Dr. Septimus Gibbon, who suggested that if the board could see their way to the concession it would be well to alter the rules so that a member of the staff, if also a governor, might be able to vote at a general meeting. He also paid a warm tribute to Mr. Rawlings as an administrator.

Mr. Melville Green thought that if the motion were lost or carried, it would be alike unfortunate. On the one hand they would be threatened with the resignation of the staff, which, though they no more than any man or body of men were indispensable, would yet be a great calamity; and on the other hand the board, though he had heard no suggestion as to their resignation, would regard the loss of the motion as a vote of censure. He was bound to say he thought the board were greatly exaggerating the evils that might arise from the presence of two members of the staff on the governing body. He thought perhaps the secretary-director, though exceedingly devoted to the interests of the hospital, had not displayed as much tact as might have been desired, or the rupture would not have reached its present point. He suggested that they might find a way out of the difficulty and provide a golden bridge if, instead of voting on the resolution before them, they took up the second point and asked a committee of, say, five or seven eminent gentlemen to investigate and report on the whole matter. No doubt either side would be willing to accept their ruling. Personally he sympathised with both sides, but if he had to vote on this resolution he must vote against the board.

Sir Henry Burdett said the last speaker had taken up the position referred to by Lord Crewe last week, and preferred to sit on the fence, since whichever side he came down he thought he would get his feet in a mess. But he assured him there was no mess, and there was no doubt they ought to come to a decision. There were three distinct ways of hospital government. The first was where the whole of the honorary staff were *ex-officio* members of the board. The second was for two members of the staff to be annually elected by their colleagues to sit on the board. The third was where the medical staff recognised that their duties were professional, and preferred to maintain their independence by having a medical committee of their own, to whom were referred all questions, so that they had an opportunity of giving the opinion of the majority of the staff, and this invariably prevailed with the management. This was held by many to be the sounder and wiser course, and at the great hospitals where it was in vogue it worked for absolute efficiency, and peace prevailed. In view of the trouble they had been going through in connection with the War Office, where there were both civilian and military heads, he thought that any hospital which, like the National, was free from dual control, ought to hesitate a long time before they introduced the system. But since the staff thought it was not well to work smoothly through an intermediary official, even when that official was so able a one as Mr. Burford Rawlings, he thought the board might take steps to remove the difficulty in a perfectly constitutional manner. The rules provided that the Medical Committee should consist, in addition to the physicians and surgeons, of two members of the board of management elected by the board. If this provision, which he understood had fallen into abeyance of late years, were put into force, it would promote personal intercourse and harmony. If the practice had been kept up, he thought they might not have had to be there that day. In another matter he was glad to learn that Mr. Russell agreed with the suggestion of Dr. Gibbon, namely, that any officer who had the means or the interest to qualify as a governor should have full representation at general meetings. It was a very usual rule. It was quite possible that these points might have given rise to friction and that their removal would help to put things right. But as regarded efficiency he emphatically recorded his deliberate opinion that the hospital was without doubt a splendid one, and a credit to the metropolis of the Empire. Mr. Holland and himself had carefully gone over it, and they both felt that it was so administered that from top to bottom there was the maximum of efficiency, of cleanliness, and of loyalty and contentment on the part of the nursing staff, and that looked at generally it was a worthy example of the highest unit of efficiency and usefulness. Referring to Mr. Russell's mention of the fear of encouraging vivisection being quoted as a reason for withholding subscriptions, Sir Henry said people always had twenty good reasons why they should keep their money in their pockets. But that apprehension of being experimented upon was not the general attitude was proved by the fact that every year the number of people who wanted to be treated in the hospitals increased out of all proportion to the increase in the population; in fact, the hospitals were growing almost too popular. As to the purpose for which they were met, it was necessary to come to a decision, and he hoped they would support the board, and that in future, with the modifications suggested, there would be harmonious working under the system which existed, and which existed also in so many of their best hospitals

The Chairman and Mr. Pearman both expressed the belief that the board would be quite willing to revert to the practice of members of the board being on the Medical Committee, which had only dropped into desuetude at the desire of the committee. They were willing, Mr. Russell said, to cordially pass an act of forgetfulness and to welcome a resumption of harmonious relations. He mentioned that four members of the medical staff were governors, and could have attended and spoken at the meeting had they wished, apart from the invitation to speak he had then extended to any of them present.

On the vote being taken only two hands were held up against the resolution, which was, therefore, carried. It was mentioned that the board held 170 proxies.

Mr. Burford Rawlings, in reference to the remark that he had been wanting in tact, said the only case in which he had come into collision with a member of the staff was in consequence of his refusal to sign an order for the admission of a child who was obviously an idiot, this being clearly in contravention of the rules.

No other motion being brought forward, the proceedings terminated with a vote of thanks to the chairman, moved by Mr. Melville Green.

